# Pharmacokinetic study of two different rifabutin doses co-administered with lopinavir/ritonavir in African HIV and tuberculosis co-infected adult patients

**DOI:** 10.1186/s12879-020-05169-2

**Published:** 2020-06-26

**Authors:** Seni Kouanda, Henri Gautier Ouedraogo, Kadari Cisse, Tegwinde Rebeca Compaoré, Giorgia Sulis, Serge Diagbouga, Alberto Roggi, Grissoum Tarnagda, Paola Villani, Lassana Sangare, Jacques Simporé, Mario Regazzi, Alberto Matteelli

**Affiliations:** 1 Biomedical and Public Health Department, Institut de Recherche en Sciences de la Santé (IRSS), Ouagadougou, 03BP7192 Burkina Faso; 2 Department of Epidemiology, Biostatistics and Occupational Health, McGill University, Montreal, QC Canada; 3 McGill International TB Centre, McGill University, Montreal, QC Canada; 4 Institute of Infectious and Tropical Diseases, Brescia University Hospital, Brescia, Italy; 5 Institute of Pharmacology, IRCCS, San Matteo University Hospital, Pavia, Italy; 6 Yalgado Ouedraogo University Teaching Hospital, Ouagadougou, Burkina Faso; 7 Centre de Recherche Biomoléculaire Pietro Annigoni (CERBA), Ouagadougou, Burkina Faso

**Keywords:** Pharmacokinetic, Rifabutin, Lopinavir, HIV/tuberculosis co-infection, Burkina Faso

## Abstract

**Background:**

This study aimed to assess the pharmacokinetic profile of 150 mg rifabutin (RBT) taken every other day (every 48 h) versus 300 mg RBT taken every other day (E.O.D), both in combination with lopinavir/ritonavir (LPV/r), in adult patients with human immunodeficiency virus (HIV) and tuberculosis (TB) co-infection.

**Methods:**

This is a two-arm, open-label, pharmacokinetic, randomised study conducted in Burkina Faso between May 2013 and December 2015. Enrolled patients were randomised to receive either 150 mg RBT EOD (arm A, 9 subjects) or 300 mg RBT EOD (arm B, 7 subjects), both associated with LPV/r taken twice daily. RBT plasma concentrations were evaluated after 2 weeks of combined HIV and TB treatment. Samples were collected just before drug ingestion and at 1, 2, 3, 4, 6, 8, and 12 h after drug ingestion to measure plasma drug concentration using an HPLC-MS/MS assay.

**Results:**

The Cmax and AUC_0–12h_ medians in arm A (Cmax = 296 ng/mL, IQR: 205–45; AUC_0–12h_ = 2528 ng.h/mL, IQR: 1684–2735) were lower than those in arm B (Cmax = 600 ng/mL, IQR: 403–717; AUC_0–12h_ = 4042.5 ng.h/mL, IQR: 3469–5761), with a statistically significant difference in AUC_0–12h_ (*p* = 0.044) but not in Cmax (*p* = 0.313). No significant differences were observed in Tmax (3 h versus 4 h). Five patients had a Cmax below the plasma therapeutic limit (< 300 ng/mL) in the 150 mg RBT arm, while the Cmax was above this threshold for all patients in the 300 mg RBT arm. Additionally, at 48 h after drug ingestion, all patients had a mycobacterial minimum inhibitory concentration (MIC) above the limit (> 64 ng/mL) in the 300 mg RBT arm, while 4/9 patients had such values in the 150 mg RBT arm.

**Conclusion:**

This study confirmed that the 150 mg dose of rifabutin ingested EOD in combination with LPV/r is inadequate and could lead to selection of rifamycin-resistant mycobacteria.

**Trial registration:**

PACTR201310000629390, 28th October 2013.

## Background

Tuberculosis (TB) is the most important AIDS-related opportunistic disease globally and is the leading cause of death among people living with HIV, accounting for an estimated 25% of such deaths [1]. Conversely, over 23% of people who die from TB are also infected with HIV. The introduction of antiretroviral treatment (ART) has had a significant impact on the reduction of TB incidence and mortality at both the individual and population levels over the past two decades [2–6]. Rifamycins (rifampicin, RIF or rifabutin, RBT) represent the core component of conventional anti-TB treatment regimens, even in combination with ART. RBT is a less potent inducer of CYP3A4 compared to RIF [7–11]. It is recommended for prophylaxis and treatment of *Mycobacterium avium complex (MAC)* as well as for treatment of drug-susceptible TB [11]. The 25-O-desacetyl-rifabutin metabolite contributes up to 10% of the total anti-bacterial activity [12, 13].

The clinical management of TB/HIV co-infection can be quite challenging for a number of reasons, including important drug interactions between rifamycins and protease inhibitors (PIs) [14–16]. PIs are well-known CYP3A inhibitors that lead to the accumulation of substrates such as RBT [17]. The standard RBT dose consists of 300 mg administered once daily and is to be reduced to 150 mg every other day if in combination with a PI [12, 18], although some experts recommend a double dose (i.e., 300 mg every other day) [19]. The US guidelines for TB treatment in HIV-infected adults now recommend the administration of rifabutin at a daily dosage of 150 mg with a boosted protease inhibitor [20]. The rationale for RBT dosage reduction lies in the attempt to limit its side effects, though this strategy is currently supported by very little evidence [21, 22].

In humans, RBT is rapidly absorbed, and peak plasma concentrations are reached within 2–4 h after oral administration. After a single dose of 300, 450 or 600 mg in healthy volunteers, the pharmacokinetics of RBT is linear.

In HIV infected subject, RBT peak plasma concentrations are reached within 2–4 h after oral administration. However it is important to point out that given the high interindividual variability of (RBT oral drug absorption, single time points may miss the actual peak concentrations and provide poor information about drug absorption status [23] . RBT has a large volume of distribution, reaching several organs except for the brain [12, 24]. Importantly, 24 h after administration, the drug levels in human lung tissue are 5–10 times higher than plasma levels. The intracellular penetration of RBT is very high, as evidenced by reports of intracellular/extracellular penetration ratio in humans from 9:1 to 15:1 in neutrophil and monocytes. The high intracellular concentration likely plays a crucial role in determining the efficacy of RBT against intracellular pathogens such as mycobacteria.

Clinical trial data on the pharmacokinetics of RBT administered in combination with lopinavir/ritonavir (LPV/r) are very scarce in Africa [11], but studies conducted in Europe and North America suggest that the currently recommended alternate day dosing might be inadequate to effectively treat the co-infection, thus requiring close monitoring of RBT plasma concentrations in HIV-positive patients [8, 10].

The optimum pharmacokinetic parameter associated with antitubercular treatment efficacy is unknown, Nevertheless, results of various studies suggest that achievement of the target serum levels for first-line anti-TB drugs is an important objective. Target concentrations of these agents have been proposed based on the concentrations achieved in healthy volunteers and patients receiving the standard doses [23, 25–27].

Studies in which serial blood samples were withdrawn to characterize not only Cmax but also the AUC of rifabutin are relatively few among African adult patients. Rifabutin reasonable therapeutic ranges based on peak plasma concentrations (Cmax) have emerged (19) even if precise, validated targets for peak plasma concentrations of this agent relative to their minimal inhibitory concentrations (e.g., Cmax/MIC) are not available from human studies [28]. Rifabutin Cmax lower than 300 ng. mL were identified as at the highest risk of ARR (Acquired Rifamycin Resistance), while Cmax higher than 900 ng/ml may be warranted and increase the risk of leucopenia, skin discoloration, arthralgias, and anterior uveitis [23, 29]. If patients can achieve ‘normal’ serum concentrations, then poor drug absorption is not the reason for the poor clinical responses; a search for other causes can be continued with greater confidence.

The technical platforms for pharmacokinetic monitoring are largely unavailable in most African countries, which contributes to the limited use of RBT in routine clinical practice. Although RIF-based anti-TB regimens are generally preferred, the increasing number of patients receiving second-line antiretroviral treatment, including protease inhibitors (PI-based ART) in Africa poses significant clinical challenges for the management of HIV and TB co-infection [30]. The identification of the optimal dose for RBT when combined with PIs and other antiretroviral drugs is therefore a key research priority. Our study aimed to assess the pharmacokinetic profile of RBT and its active metabolite 25-O-desacetyl-rifabutin (d-RBT) using two dosing regimens (150 mg or 300 mg RBT taken every other day) in TB-HIV co-infected adult patients in Burkina Faso, a resource-limited, TB- and HIV-endemic country.

## Methods

### Study design

We conducted a pilot, open-label study to assess the pharmacokinetic profile and the tolerability of two alternative dosages of RBT in combination with LPV/r in TB-HIV co-infected adult patients.

### Study population

HIV and TB co-infected patients were recruited from the TB Diagnosis and Treatment Centres (CDTs) of Bogodogo and Kossodo district hospitals in Ouagadougou (Burkina Faso) between May 2013 and December 2015. Eligible patients were male and non-pregnant/non-lactating female subjects aged 18 to 60 years who were diagnosed with HIV-1 infection (regardless of their CD4+ T cell lymphocyte count) and who had confirmed or suspected pulmonary TB. All patients had to be naïve to both anti-TB treatment and ART and eligible for LPV/r- and RBT-based regimens and had to provide written informed consent to participate in the study. Those with plasma alanine aminotransferase (ALT) levels greater than 5 times the upper limit of the normal range and/or plasma creatinine levels higher than 3 times the normal value were excluded from the study.

Pharmacokinetic studies have often been conducted on a limited number of participants, and generally there is no optimal size required [31, 32]. We had planned to include 30 TB/HIV co-infected patients for the study, (15 patients in each arm), but we could only include 16 during the study period. These patients were subsequently allocated to one of the two study arms as shown in Table 1. The allocation process consisted of simple randomisation (i.e patients were alternatively include in the treatment groups), and the allocation numbers were assigned by the study assistant to patients as they were recruited at study sites.

**Table 1:** Study treatment arms

	***Day 1 to Day 14***	***Day 15 after anti-tuberculosis initiation***	***Day 14 after combined treatment (anti-TB and ART)***
**arm**	**Anti-tuberculosis alone**	**Combined anti-tuberculosis and ART** **(for the duration of anti-TB therapy)**	**Pharmacokinetic monitoring**
A	“300 mg rifabutin + standard ethambutol-isoniazid-pyrazinamide regimen” daily for 14 days	150 mg rifabutin **every other day** + standard ethambutol-isoniazid-pyrazinamide regimen combined with ART regimen including 200/50 mg lopinavir/ritonavir taken twice daily	Blood taken for pharmacokinetic monitoring
B	“300 mg rifabutin + standard ethambutol-isoniazid-pyrazinamide regimen” daily for 14 days	300 mg rifabutin **every other day** + standard ethambutol-isoniazid-pyrazinamide regimen combined with ART regimen including 200/50 mg lopinavir/ritonavir taken twice daily	Blood taken for pharmacokinetic monitoring

### Therapeutic management

Patients first started anti-TB therapy with RBT-based regimen for 14 days, before receiving antiretroviral therapy with LPV/r. The RBT dose (150 mg or 300 mg) was administered every other day (every 48 h) on an empty stomach at 7 AM, 1 h before breakfast (combined with ethambutol, pyrazinamide and isoniazid taken daily). The dose of LPV/r was taken at 8 AM (two capsules of 200/50 mg) and at 8 PM (two capsules of 200/50 mg), regardless of meals.

### Pharmacokinetic monitoring

After 15 days of combined therapy with RBT and LPV/r, patients were admitted to the hospital and fasted from midnight. The pharmacokinetic assessment was performed on the day of RBT administration.

The first pharmacokinetic monitoring measurement (time zero) was performed at steady-state on an empty stomach just before the morning dose of RBT (48 h after the previous dose). After the first blood sample, the patient immediately took (within 5 min) the RBT-containing regimen followed by the ART as specified above. Subsequent blood samples for pharmacokinetic monitoring were obtained at 1, 2, 3, 4, 6, 8 and 12 h after combined drug ingestion. Breakfast was provided to the patient upon completion of the second sampling.

At each withdrawal, 2–3 mL of blood were collected in a heparinized tube and centrifuged at 3000 rpm for 10 min within 1 h of collection. The blood plasma was stored in a refrigerator at − 20 °C until pharmacokinetic analysis.

A high-performance liquid chromatography-mass spectrometry (HPLC/MS-MS) assay previously described by Moyer et al. [33] was used to measure the concentration of RBT and its metabolite (d-RBT) plasma concentrations at the Service of Clinical Pharmacology (IRCCS Policlinico San Matteo, Pavia, Italy). The limit of quantification was 50 ng/mL. The assay was validated in accordance with the European Medicines Agency (EMA) guidelines for bio-analytical methods [34]. The areas under the plasma concentration-time curve (AUC_0–12h_) were calculated from time 0 to 12 h after drug administration by using the linear trapezoidal rule.

### Patients’ follow-up

All patients were monitored at the centres dedicated to TB/HIV co-infection management until the end of the anti-mycobacterial treatment. All patients received RBT-based treatment in place of RIF for the duration of anti-TB therapy. Anti-TB treatment was provided under direct supervision in line with the DOTS (directly observed treatment short-course) strategy [35], and the national guidelines were followed for the management of HIV infection. All patients received daily cotrimoxazole preventive therapy (CPT). Treatment adherence was assessed through self-administrered questionnairesas well as by counting the number of pills brought back to the visit by the patient. To promptly identify any drug-induced adverse events, a clinical examination was performed weekly during the first month after study entry and then monthly according to routine visits as planned for standard TB/HIV co-infected patients. Microbiological response to treatment was assessed by sputum-smear microscopy at 2 months and 5 months since TB treatment initiation. TB treatment outcome was defined as cure, treatment completed, treatment failure, and treatment success, according to WHO guidelines for monitoring of TB treatment [36].

Laboratory tests such as a blood panel and basic biochemistry (i.e., liver function tests, creatinine, amylase, bilirubin, lipid profile) were performed once every 2 weeks during the first month and then monthly. CD4 lymphocyte cell count monitoring was performed quarterly.

### Data management and analysis

Participants’ clinical and laboratory data were collected by the study investigators (physicians) using a case report form and checked by the study monitor. Data were entered in EpiData (http://www.epidata.dk) and Excel (Microsoft) spreadsheets. Data analyses were performed using Stata statistical software version 13 (StataCorp LP; College Station, TX, USA). Frequency measures, proportions and median values were used to describe the patients’ characteristics. For the pharmacokinetic profile, the following parameters were determined for each patient at steady-sate: Cmax (peak plasma concentration in μg/mL), Cmin (minimum plasma concentration in ng/mL), Ctrough (concentration just before the next dose in ng/mL), Tmax (time until Cmax), AUC_0–12h_ (area under the curve, calculated as plasma concentration/time, ng x h/mL, from 0 to 12 h after drug administration). Each pharmacokinetic parameter was presented as the median with an interquartile range (IQR). Fisher’s exact test was applied to compare the two groups; all the statistical tests were two-sided, with a threshold of 0.05 for rejecting the null hypothesis.

### Ethics

The study protocol was approved by the National Ethics Committee for Health Research (deliberation N°2012–5-031) and registered in the Pan African Clinical Trial Registry with number PACTR201310000629390. Participation in the study was voluntary following the signing of informed consent. All patients were treated free of charge, including all medications and biochemical tests. Transportation costs related to the study were also reimbursed to all study participants.

## Results

### Characteristics of the study population

**Fig. 1:**
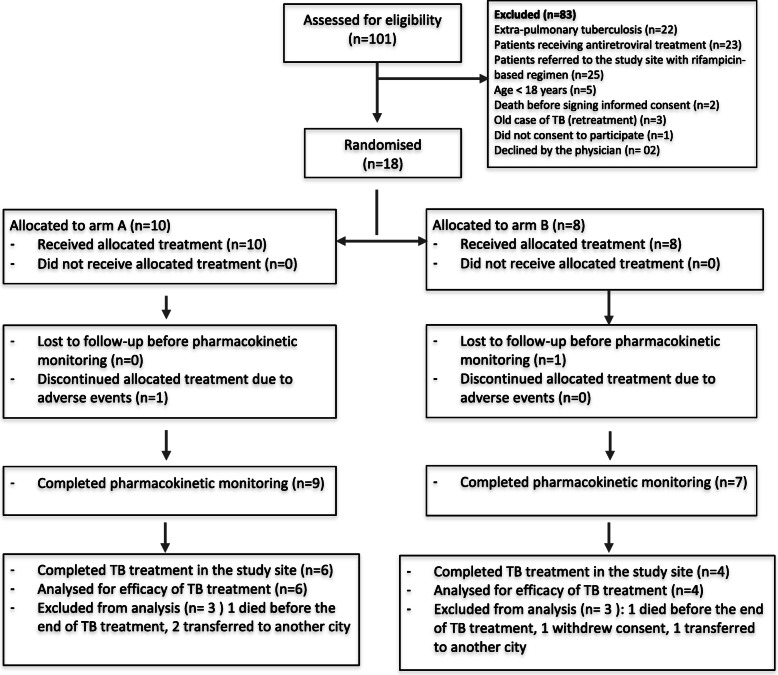
Patient Flow Chart for the study

A total of 101 patients were assessed for eligibility, and 18 of them were finally included and randomised to either arm A (*n* = 10) or arm B (*n* = 8). Figure 1 shows the flow of participants through the different stages of the study. The socio-demographic and clinical characteristics of the study population are summarized in Table 2.

**Table 2:** Patient characteristics and laboratory parameters at the study inclusion

	**Arm A** **(*n*** **= 9)**	**Arm B** **(*n*** **= 7)**	***p*value**
Patients’ characteristics	(m + SE)	(m + SE)	
Age	36.3 ± 6.70	34.7 ± 6.92	0.643
Sex			
Male	6 (75.0)	2 (25.0)	0.131
Female	3 (37.5)	5 (62.5)	
Weight (kg)	53.2 ± 8.43	49.1 ± 8.93	0.367
Body mass index (BMI)	18.6 ± 3.08	16.5 ± 2.88	0.182
Haemoglobin (g/dL)	8.4 ± 1.95	11.1 ± 3.51	0.062
Leucocytes (10^3^/mL))	5277 ± 5385	5928 ± 3304	0.783
Neutrophils (10^3^/mL))	3795 ± 3663	3638 ± 3134	0.929
Lymphocyte (10^3^/mL))	2417 ± 2224	1442 ± 485	0.277
Monocytes (10^3^/mL))	400 ± 430	271 ± 179	0.472
AST (U/L)	46.5 ± 24.23	48.2 ± 33.10	0.905
ALT (U/L)	41.2 ± 25.43	32.8 ± 22.05	0.500
Creatinine (μmol/L)	98.2 ± 22.04	93.1 ± 21.96	0.652
Total cholesterol (mmol/L) T	143.2 ± 30.30	129.8 ± 25.91	0.368
HDL cholesterol (mmol/L)	44.7 ± 23.63	42.4 ± 16.84	0.827
Amylase (U/L)	131.0 ± 71.31	92.8 ± 53.28	0.266
Total bilirubin (μmol/L)	3.3 ± 2.26	3.8 ± 2.33	0.696
Direct bilirubin (μmol/L)	1.08 ± 0.85	1.45 ± 1.56	0.618
Lymphocytes CD4+ T (cells/μL)	221.1 ± 154.75	285.8 ± 175.39	0.446
Type of tuberculosis			
SPPT	7 (50.0)	7 (50.0)	0.475
SNPT	2 (100.0)	0 (0.0)	–
WHO HIV stage			
Stage 2	1/9	0/7	0.562
Stage 3	8/9	7/7	
Opportunistic infections			
Yes	5/9	3/7	0.500
No	4/9	4/7	

### Pharmacokinetic parameters of RBT and of d-RBT after RBT and LPV/r co-administration

**Fig. 2:**
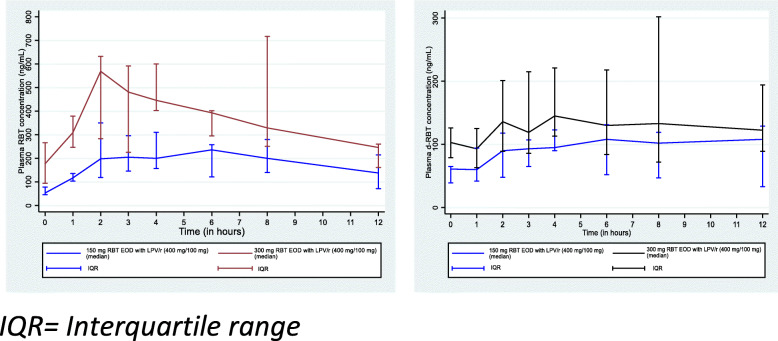
Median plasma RBT and d-RBT concentrations at specified times after the administration of RBT (150 mg or 300 mg EOD) combined with lopinavir/ritonavir (200 mg/50 mg)

The plots of median plasma concentrations of RBT and d-RBT at a specified time t after the administration of RBT are reported in Fig. 2.

Among patients in arm A, the median plasma concentrations of RBT and d-RBT before the dose administration were 53 (IQR: 46–78) ng/mL and 61 (IQR: 39–65) ng/mL, respectively, while among those in arm B, their values were 177 (IQR: 94–266) ng/mL and 103 (IQR: 79–126) ng/mL, respectively. Twelve hours after the administration of RBT, the median RBT concentration had increased to 138 (IQR: 71–215) ng/mL and 246 (IQR: 161–260) ng/mL for patients receiving 150 mg RBT EOD (every other day) and those receiving 300 mg RBT EOD (*p* = 0.460), respectively. In the 150 mg RBT EOD dosage group, the median Cmax was 296 (205–450) ng/mL, compared with 600 (IQR: 403–717) ng/mL in the 300 mg RBT EOD dosage (*p* = 0.313). The area under the curve (AUC_0–12 h_) was 2528 (IQR: 1684–2735) ng.h/mL for the dosage of 150 mg RBT EOD and 4042.5 (IQR: 3469–5761) ng.h/mL in patients receiving 300 mg RBT EOD (*p* = 0.044). The analysis of the pharmacokinetic parameters of d-RBT showed that Cmax, AUC_0–24 h_ and Tmax were higher for patients in the 300 mg RBT EOD group compared to those receiving 150 mg RBT EOD. Of note, the AUC _(0–12 h)_ of d-RBT was 1200.5 (IQR: 737.5–1295.5) ng.h/mL in patients in the 150 mg RBT EOD group and increased to 1534 (IQR: 1059.5–2351) ng.h/mL in the 300 mg RBT EOD group (Table 3).

**Table 3:** Comparison of pharmacokinetic parameters between 150 mg rifabutin and 300 mg rifabutin combined with lopinavir/ritonavir (200/50 mg)

	**150 mg RBT EOD with LPV/r (400 mg/100 mg)** **(*n*** **= 9)**	**300 mg RBT EOD with LPV/r (400 mg/100 mg)** **(*n*** **= 7)**	***p*value**
	**(Median + IQR)**	**(Median + IQR)**	
**Rifabutin (RBT)**			
Ctrough (ng/mL)	53 (46–78)	177 (94–266)	0.044
Cmax (ng/mL)	296 (205–450)	600 (403–717)	0.313
Tmax (h)	3 (2–6)	4 (2–4)	0.657
AUC_0–12 h_	2528 (1684–2735)	4042.5 (3469–5761)	0.044
Clearance			
CL (L/h)	51.5 (34.1–53.0)	23.2 (20.1–24.9)	0.044
CL (L/h/kg)	0.92 (0.63–1.02)	0.84 (0.78–1.00)	1.000
**25-O-desacetyl-rifabutin (d-RBT)**			
Ctrough (ng/mL)	61 (39–65)	103 (79–126)	0.044
Cmax (ng/mL)	129 (111–157)	160 (136–345)	0.313
Tmax (h)	6 (2–8)	3 (3–4)	0.242
AUC_0–12 h_ (ng.h/mL)	1200.5 (737.5–1295.5)	1534 (1059.5–2351)	1.000

Data are presented as medians with the range in parentheses

*RBT* rifabutin, *d-RBT* 25-O-desacetyl-rifabutin, *EOD* every other day (every 48 h), *LPV/r* lopinavir/ritonavir, *C*_*Tn*_ plasma drug concentration at a specified time, *IQR* interquartile range, *Cmax* maximum (peak) plasma drug concentration, *Tmax* Time to reach maximum (peak) plasma concentration following drug administration, *C0* trough plasma concentration (measured concentration at the end of a dosing interval at steady state (48 h) [taken directly before next administration]), *AUC*_*t0–12h*_ area under the plasma concentration-time curve within time span t0 to t2

The ratios of the geometric mean of rifabutin and 25-O-desacetylrifabutin are presented in Table 4. There was an almost 50% increase in Cmax and AUC_0–12h_ in the RBT 300 mg EOD arm compared to the RBT 150 mg EOD arm. The same finding was noted for the same parameters with 25-O-desacetylrifabutin. However, the changes in Tmax were not significant between the two arms.

**Table 4:** Geometric means ratio of plasma RBT and d-RBT concentrations after the administration of RBT (150 mg or 300 mg EOD) combined with lopinavir/ritonavir (200 mg/50 mg)

**Plasma RBT concentration (ng/mL)**	**GMR (150 mg RBT/300 mg RBT)**	**95%CI**		***p*value**
Cmax	0.473	0.264	0.847	0.015
Tmax	0.841	0.464	1.522	0.540
AUC_0–12h_	0.510	0.315	0.823	0.009
**Plasma d-RBT concentration (ng/mL)**				
Cmax	0.544	0.303	0.977	0.042
Tmax	1.254	0.615	2.559	0.507
AUC0–12	0.597	0.333	0.830	0.009

Abbreviations: *GMR* geometric means ratio, *RBT* rifabutin, *d-RBT* 25-O-desacetyl-rifabutin, *EOD* every other day

### Analysis of individual pharmacokinetic profiles in both groups

The analysis of individual plasma concentrations of RBT at steady-sate showed that just before ingestion of the RBT dose (i.e., 48 h after ingesting 150 mg or 300 mg RBT), all patients had a plasma concentration of RBT (Ctrough) above the therapeutic limit (> 300 ng/mL). This concentration was greater than the MIC (60 ng/mL) in all patients taking 300 mg RBT EOD, while 5 out of 9 patients taking 150 mg RBT EOD had a Ctrough below this threshold.

### TB treatment outcome and immunological response

Of the 15 patients (14 sputum smear-positive and 1 sputum smear-negative) who underwent microbiological testing 2 months after TB treatment initiation, 14 were sputum smear-negative. The only patient who was still sputum smear-positive belonged to the 150 mg RBT EOD group and remained positive at 5 months after enrolment in treatment. All the other patients were followed up until the end of the anti-TB treatment regimen and declared cured or treatment completed in accordance with international standards [36].

During the study period, an increase in the mean CD4 lymphocyte cell count was observed in patients from both treatment groups: from 221.1 ± 154.75 cells/μL to 581 ± 299 cells/μL in arm A and from 285.8 ± 175.39 cells/μL to 505 ± 53 cells/μL in arm B.

### Adverse events

Our study was not powered to determine the safety profile of RBT-containing regimens. However, it is important to point out that a total of 5 severe adverse events (SAEs) were observed during the study period (Table 5). One patient from arm A and one patient from arm B died 4 and 5 months after treatment initiation, respectively, although it is unlikely that these deaths were related to treatment. In fact, both patients had a very low CD4+ T cell count at baseline (25 cells/mL and 98 cells/mL), were hospitalized for severe anaemia (7 days after anti-TB treatment initiation) and received blood transfusion; additionally, they were both sputum smear-negative at 2 months.

**Table 5:** Proportion and grade of most frequent adverse events observed in each arm of the study

**Clinical**	**Arm A**					**Arm B**				
	**Adverse Events**	Grade				**Adverse Events**	Grade			
		Grade 1	Grade 2	Grade 3	Grade 4		Grade 1	Grade 2	Grade 3	Grade 4
Asthenia/fatigue	5/9	4	1			3/7	2	1		
Headaches	4/9	3	1			6/7	5	1		
anorexia	7/9	7				3/7	3			
diarrhoea	1/9	1	1			2/7	2			
Nausea/vomiting	1/9	1				3/7	3			
Arthralgia	1/9			1		5/7	1	3	1	
Insomnia	6/9	4	2			3/7	2	1		
vertigo	5/9	4	1			0/7				
Skin rash	5/9	4	1			2/7	1	1		
Death	1/9				1	1/7				1
**Laboratory**										
Anaemia	9/9	2	3	2	2	7/7	3	2	1	1
Leukopenia	3/9	3				1/7	1			
Neutropenia	1/9	1				2/7	1	1		
Thrombopenia	0/9					1/7		2		
ALT elevated	6/9	4	2			0/7				
ASAT elevated	7/9	6	1			3/7	3			
Amylasemia elevated	3/9	3				2/7		2		
Bilirubinemia T elevated	4/9	4				5/7	3	2		

The other SAEs included one case of grade 3 peripheral neuropathy that led to the discontinuation of ART 22 days after initiation and a case of ascites with pleural effusion at 3 months of treatment (Table 5). The medical team could not establish any links between these events and the ongoing treatment. In contrast, one patient in the 300 mg RBT EOD group experienced moderate unilateral uveitis (right eye) likely induced by RBT 4 months after treatment, requiring ophthalmologic consultation and ambulatory monitoring.

Several minor side effects (grade 1) such as mild anaemia, alterations of liver function tests, and hyperbilirubinemia were observed at baseline and in both study arms. Two patients in arm B also had grade 2 haematological abnormalities (neutropenia, thrombopenia) and two other patients had grade 2 hyperbilirubinemia. No grade 3 or 4 biochemical adverse effects were observed (Table 5). Most of these effects were observed after 2 to 6 weeks of anti-TB treatment.

## Discussion

In our study serial blood samples were withdrawn to characterize accurately not only Cmax but also the AUC_0–12h_ of RBT, and to be able to compare the drug systemic exposure related to the two dosing regimens adopted. The study results showed a significant difference in Cmax and AUC_0–12 h_ between two different dosage regimens (150 versus 300 mg) of RBT, both administered every other day in combination with LPV/r-based ART.

The concentrations of the 25-O-desacetyl-rifabutin metabolite (d-RBT) were not statistically different between the 2 arms except for the Ctrough values, presumably, due to a high variability in the rate of formation and to the short time period of plasma concentrations monitoring (12 h) relatively to the RBT dosage interval (24 h) [29].

Our findings are consistent with those reported in other similar studies conducted elsewhere [11, 37, 38]. For instance, research from South Africa highlighted that the peak concentrations of RBT were significantly reduced in patients taking 150 mg RBT EOD associated with LPV/r [11].. Additionally, the Cmax of RBT administered at a dosage of 150 mg EOD were 36%, which is less than what observed with daily administration of 300 mg RBT in a study conducted in Japan [37]. Although there is no evidence yet in support of either dose of RBT taken EOD in combination with LPV/r, the intermittent administration of rifamycin-containing treatments has often been associated with an increased risk of failure and the emergence of drug-resistant mycobacterial strains [28–30].

According to our findings, the C_trough_ plasma concentration of RBT (at the end of the 48 h dosage interval remained above the therapeutic limit (300 ng/mL) in all patients from both study arms, but it was below the MIC (64 ng/mL) [39] in 5 patients taking 150 mg RBT EOD, one of whom was sputum smear-positive at two and 5 months of TB treatment.

Sputum culture could not be performed to confirm the diagnosis of TB and monitor treatment outcomes due to the lack of resources and laboratory infrastructures. Furthermore, because of the unavailability of drug susceptibility tests both at enrolment and at the end of follow-up, it was not possible to investigate the causes of treatment failure for the only patient who lacked sputum conversion.

It should be noted, however, that RBT is generally effective against *M. tuberculosis*. In fact, RBT distributes widely throughout the body, it has been detected in all tissues and body fluids examined and readily penetrates leukocyte cell membranes [15, 40, 41]. In addition, its active metabolite (d-RBT) significantly contributes to the anti-mycobacterial activity of RBT [12]. These features may explain the success of treatment in patients who have low plasma concentrations of rifabutin in the 150 mg RBT EOD group [37].

RBT is an effective alternative to RIF when drug-drug interactions are an issue. Its pharmacological features such as the large volume of distribution, the ability to penetrate across a wide range of tissues and body fluids and the fact that its main metabolite (d-RBT) contributes up to 10% of the total activity certainly promote the anti-mycobacterial efficacy of the molecule and might explain the treatment success of patients receiving lower dosages (i.e. 150 mg RBT EOD) [12, 15, 37, 41].

The small sample size and the lack of culture capabilities in our study did not allow us to establish a relationship between RBT plasma concentrations and the rate of treatment success. However, we believe that under-dosage could explain the failure outcome observed in one patient receiving 150 mg RBT EOD. In contrast, all patients receiving 300 mg RBT EOD were reported as cured or treatment completed.

Most of the studies that evaluated the 150 mg RBT EOD in TB-HIV co-infected patients concluded that RBT levels in these patients were very low [16–18, 21, 22, 27], with potential for failure of TB treatment. Conversely, a daily dose of 150 mg RBT was relatively well tolerated and more likely to reach target RBT concentrations than 150 mg RBT EOD [11]. To limit the emergence of resistance to rifamycins that could be associated with low concentrations of RBT, the CDC guidelines currently recommend a daily dose of 150 mg RBT when administered with PIs in adults, though these guidelines are based on little evidence [42].

However, we believe that 300 mg RBT EOD might be a better tolerated alternative that could limit the emergence of adverse events such as neutropenia, and thrombopenia a quite common occurrence in TB/HIV patients receiving concomitant RBT and ART which is likely dose-related [11, 16, 43, 44]. RBT-associated uveitis is another important though rare adverse event that requires special attention and necessitates drug discontinuation, but a dose-response relationship has yet to be defined [45, 46].

Additionally, an every other day administration could potentially lead to better treatment adherence compared to daily regimens, especially in patients who take several medications due to comorbidities such as those with TB and HIV co-infection. This could be particularly important in low-resource settings where there is limited capacity for long-term daily patient monitoring. However, a deeper investigation of such aspects of care was beyond the scope of our study.

Our study did not evaluate the virologic success of antiretroviral therapy, but the CD4 lymphocyte cell count analysis showed that both dosages of RBT (150 mg EOD and 300 mg EOD) combined with LPV/r had a significant beneficial effect on the immunological goal. Also, we could not evaluate the potential effects on different RBT regimens on varying dosages of LPV/r such as super-boosted lopinavir (i.e. twice daily LPV/r 400/400 mg) or double dose LPV/r. Given the pharmacokinetics of both RBT and PIs, we could expect an even greater reduction in RBT concentration if higher doses of LPV/r are used, which would contribute to favour the 300 mg RBT EOD regimen as opposed to the 150 mg RBT EOD one.

## Conclusion

In conclusion, our study suggests that 150 mg RBT administered thrice weekly in association with LPV/r for the treatment of HIV-TB co-infection might be inadequate and could lead to the emergence of rifamycin-resistant mycobacterial strains. However, a higher dosage (i.e., 300 mg RBT EOD) could increase the risk of toxicity in co-infected patients. Further studies are required to identify the optimal dosing schedule of RBT and to better assess whether a daily dose of 150 mg RBT could be sufficient to reach the appropriate plasma concentrations in patients receiving PIs and other antiretroviral drugs.

## Data Availability

The datasets used during the current study are available on request to Seni Kouanda, senikouanda@gmail.com .

## Abbreviations

AIDS: Acquired Immune Deficiency Syndrome
ART: Antiretroviral therapy
ARV: Antiretroviral
AUC: Area Under the Curve
CDC: Centers for Disease Control and Prevention
Cmax: Maximum concentration
DOTS: Directly observed treatment short-course
d-RBT: 25-O-desacetyl-rifabutin
EOD: Every other day
HIV: Human Immunodeficiency Virus
IQR: Interquartile range
LPV/r: Lopinavir/ritonavir
PIs: Protease inhibitors
RIF: Rifampicin
RBT: Rifabutin
TB: Tuberculosis
Tmax: Time at which the Cmax is observed
